# Safety and efficacy of vemurafenib in end stage renal failure

**DOI:** 10.1186/1471-2407-13-581

**Published:** 2013-12-06

**Authors:** Mahesh Iddawela, Sarah Crook, Leah George, Amit Lakkaraju, Nihal Nanayakkara, Roland Hunt, William R Adam

**Affiliations:** 1 Goulburn Valley Health, Graham Street, Shepparton 3630, Australia; 2 Rural Health Academic Center, University of Melbourne, Graham Street, Shepparton 3630, Australia; 3 Goulburn Valley Imaging, Graham Street, Shepparton 3630, Australia

**Keywords:** Renal excretion, Metastatic melanoma, Vemurafenib

## Abstract

**Background:**

Serine-threonine inhibitors, such as vemurafenib, are being used increasingly in cancer treatment, and the toxicity and therapeutic benefit need to be balanced carefully both before and during treatment.

**Case presentation:**

A patient with metastatic melanoma and end stage renal failure who was on peritoneal dialysis was treated with the serine-threonine kinase inhibitor, vemurafenib. After 5 months of treatment, a substantial response to vemurafenib was observed using imaging, but when he developed a prolonged QTc interval (common toxicity criteria (CTC) grade 3), treatment was interrupted. Vemurafenib was restarted at a reduced dose when the QTc interval returned to normal. The patient has had a significant response to vemurafenib and continued on treatment for 12 months after beginning the therapy.

**Conclusion:**

This is the first reported case of end stage renal failure in a patient who is taking vemurafenib. Although the patient developed QTc prolongation, it appears to be asymptomatic, and was managed with dose reduction. This case highlights the need for closer QTc monitoring at the start and during treatment.

## Background

Metastatic melanoma is a malignancy that is associated with a poor prognosis and until recently, few treatment options were available. Vemurafenib (Zelboraf™, Roche Pharmaceuticals Ltd, Sydney, Australia), is a serine-threonine kinase BRAF inhibitor that has demonstrated efficacy in treating metastatic or unresectable metastatic melanoma that has a known mutation in BRAF protein [1]. Approximately 40–60% of cutaneous melanomas carry a BRAF mutation, which is known to enhance cell proliferation and tumor progression [2]. Vemurafenib acts as a potent inhibitor of BRAF-mediated cell signaling and proliferation, and has produced improved progression-free and overall survival in previously untreated metastatic melanoma containing BRAF V600E and V600K mutations. Vemurafenib is highly protein bound (>99%), and is excreted *via* feces (94%) and urine (1%) [3]. While it has been demonstrated that drug pharmacokinetics are not significantly altered by mild to moderate renal dysfunction, there have been no studies in patients with severe renal dysfunction. The other important consideration in patients treated with serine-threonine kinases is the effect that the renal failure has on cardiac function and serum electrolytes. Here, we report a case of a patient with end stage renal disease who was treated with vemurafenib and who developed a prolonged QTc interval during treatment that was successfully managed using dose reduction.

## Case presentation

A 50-year old male, seen by the surgical team, had a pigmented lesion on his scalp that had recently increased in size, and become tender and ulcerated. Initial excision revealed a nodular invasive malignant melanoma with a Breslow thickness of 10 mm, 5 mitosis per square millimeter, and no lymphovascular invasion, and the excision was deemed incomplete. Following this, he underwent a wide local excision and sentinel lymph node biopsy. Because two out of four lymph nodes from the left supraclavicular fossa had malignant melanoma micro-metastases, surgical dissection of the left neck nodes was performed. This showed that 3 out of 29 lymph nodes contained metastatic malignant melanoma without any extra-nodal spread, and mutation testing showed the BRAF V600K mutation.

Four years before, the patient had been diagnosed with chronic renal failure believed to be due to uncontrolled hypertension. This patient had been on continuous ambulatory peritoneal dialysis since the diagnosis, and his renal function and electrolytes were stable (plasma urea and creatinine ranged from 20–30 mmol/L and 1004–1483 umol/L, respectively, potassium was 5.2 mmol/L, calcium was 2.4 mmol/L, and magnesium was 1.02 mmol/L). There was no other significant medical history of relevance.

Three months later, a computerized tomography (CT) scan showed convincing evidence of metastatic disease with confluent lymphadenopathy in the paratracheal group of nodes, with the target node measuring 22 mm. There were also new lymph nodes in the subcarinal region, right para-oesophageal lymphadenopathy and a right lower lobe target mass. The lactate dehydrogenase (LDH) level was also elevated (526 U/L) and the patient was started on vemurafenib at the recommended dose (960 mg twice daily).

Four weeks after beginning treatment, his LDH returned to normal, and the patient denied any significant toxicities. Results of his serial electrocardiograms (ECGs) were normal. Importantly, the patient’s renal function remained stable throughout this time. Four months after beginning treatment, there was a reduction in size of the paratracheal and subcarinal nodes and the pulmonary mass was not seen. Throughout this time, the patient remained well, reporting grade 1 photosensitivity as the only side effect of treatment.

**Figure 1:**
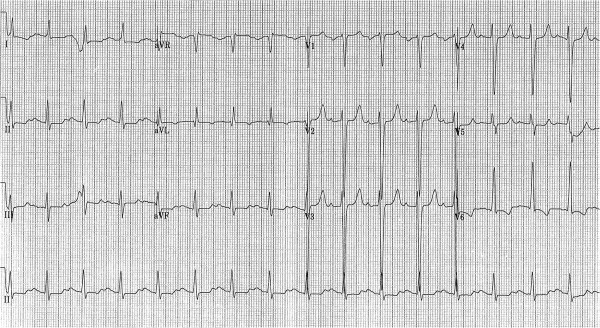
Twelve lead ECG showing the prolonged QTc interval (513 msec).

**Figure 2:**
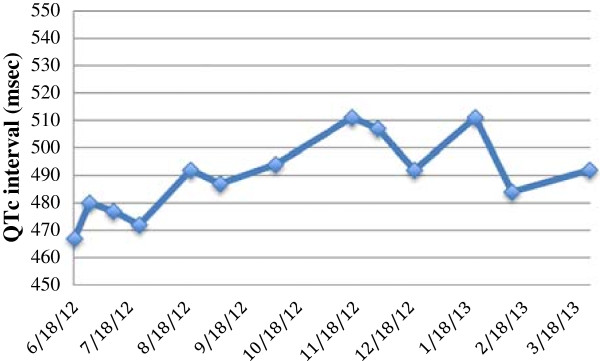
**Change in QTc interval during treatment with vemurafenib.** The QTc interval was normal at baseline and was high in November 2012. The QTc interval then returned to normal in December 2012. It also increased above 500 msec in January 2013, and has since decreased to <500 msec.

After treatment for 5 months, an ECG demonstrated that the QTc interval was increased at 511 msec (CTC grade 3) compared with baseline (467 ms), but it was still less than the baseline QTc interval of 60 ms (Figures 1 and 2). Vemurafenib treatment was stopped and other possible causes of the prolonged QTc interval were investigated. There were no changes in his renal function, electrolyte levels were normal and he was not on any new medications. A 24-h Halter monitor assessment was carried out to check for the presence of any arrhythmias or any periods of torsade de pointes, but none were found. Serial ECGs were carried out, and the QTc returned to the baseline level over 3 weeks. The patient was re-started on vemurafenib at a reduced dose (720 mg BD) after discussion with both the renal and cardiology teams. Throughout treatment, the patient’s renal function remained stable and there were no significant variations in the electrolytes. In addition, it was noted that the patient had also had a high QTc interval (512 msec) 2 years previously, which resolved spontaneously and no precipitating cause was found, and there were no other documented episodes of a prolonged QTc interval.

This patient continued on the reduced dose of vemurafenib, and after one month, his QTc interval again increased to 511 ms, but because it was still less than 60 ms from baseline, a dose interruption was deemed unnecessary because he had an increased QTc interval before starting treatment. An additional ECG a week later confirmed that the QTc interval was normal and the fluctuation was not associated with any symptoms. Staging scans 12 months after re-initiating the treatment showed that the patient had a partial response according to RECICT criteria.

This is one of the first reported cases where vemurafenib was administered to a patient with chronic renal failure and dialysis. The case demonstrates some important aspects about the use of serine-threonine kinase inhibitors in patients with cancer and other co-morbidities. There is limited data on vemurafenib in patients with severe renal impairment, but because hepatic metabolism is the main route of drug excretion, it is theoretically safe [4]. This report shows that other factors need to be considered in this context because electrolyte abnormalities or cardiac co-morbidities could lead to potential complications.

Management of patients with changes in the QTc interval is an important issue that needs to be evaluated because this class of drugs is being increasingly used. The QTc interval is prolonged in a dose-dependent manner in response to vemurafenib, and the risk of malignant arrhythmias such as torsade de pointes can occur with any increase in QTc interval, even though the risk is higher at longer intervals [5]. Cardiovascular disease remains the most common cause of death among patients with end stage renal failure and cardiac arrhythmias are an important contributor [6]. One of the major difficulties in measuring the QTc interval is diurnal variation, and prolonged QTc intervals are seen in patients with renal failure for many reasons. The common causes of QTc changes in patients with renal failure include electrolyte disturbances, cardiac fibrosis and hypertrophy, changes in cellular or interstitial composition during dialysis, and iron overload. The patient had one episode of prolonged QTc interval 2 years prior to this reported event. However, there was no further documented evidence of QTc abnormalities and because a prolonged QTc interval is a potential drug side effect that can have significant consequences, vemurafenib was initially considered to have caused of the prolongation. However, a detailed adverse drug reaction analysis using established criteria revealed that the probability of this being a drug reaction was low [7]. The patient was started on lower dose as a precaution.

The risks and benefits need to be carefully assessed on a case-by-case basis. The decision was made by the team to continue vemurafenib treatment in this patient because the patient was responding and the adverse event risk was low, even though the QTc interval increased to > 500 ms from <60 ms at baseline.

## Conclusions

This case study shows that vemurafenib can be used safely in patients with chronic renal failure. It also highlights the importance of regular follow-up including serial ECGs to prevent treatment complications that result from other co-morbidities, such as a prolonged QTc interval. It also demonstrates that such changes may occur after several months of treatment, and it is important to investigate all possible causes including those related to other co-morbidities.

## Consent

Written informed consent was obtained from the patient for publication of this case report and any accompanying images. A copy of the written consent is available for review by the Editor of this journal.

## Competing interests

The authors declare that they no competing interests.

## Authors’ contributions

MI was involved in manuscript preparation, literature search and patient care. SC and LG were involved in manuscript preparation and patient care. RH, AL, NN and WRA were involved in a patient care, manuscript preparation and review. All authors read and approved the final manuscript.

## Pre-publication history

The pre-publication history for this paper can be accessed here:

http://www.biomedcentral.com/1471-2407/13/581/prepub

## References

[B1] Chapman PB Hauschild A Robert C Improved survival with vemurafenib in melanoma with BRAF V600E mutation NEJM 2011 13 26 2507 2516 10.1056/NEJMoa1103782 21639808 PMC3549296

[B2] Davies H Bignell GR Cox C Mutations of the BRAF gene in human cancer Nature 2002 13 6892 949 954 10.1038/nature00766 12068308

[B3] Heakal Y Kester M Savage S Vemurafenib (PLX4032): an orally available inhibitor of mutated BRAF for the treatment of metastatic melanoma Ann Pharmacother 2011 13 11 1399 1405 10.1345/aph.1Q363 22028422

[B4] Flaherty KT Yasothan U Kirkpatrick P Vemurafenib Nat Rev Drug Discov 2011 13 11 811 812 10.1038/nrd3579 22037033

[B5] Voiculescu M Ionescu C Ismail G Frequency and prognostic significance of QT prolongation in chronic renal failure patients Rom J Intern Med 2006 13 4 407 417 18386617

[B6] Brell JM Prolonged QTc interval in cancer therapeutic drug development: defining arrhythmic risk in malignancy Prog Cardiovasc Dis 2010 13 2 164 172 10.1016/j.pcad.2010.05.005 20728704 PMC2956432

[B7] Naranjo CA Busto U Sellers EM A method for estimating the probability of adverse drug reactions Clin Pharmacol Ther 1981 13 2 239 245 10.1038/clpt.1981.154 7249508

